# Putative Antimicrobial Peptides of the Posterior Salivary Glands from the Cephalopod *Octopus vulgaris* Revealed by Exploring a Composite Protein Database

**DOI:** 10.3390/antibiotics9110757

**Published:** 2020-10-30

**Authors:** Daniela Almeida, Dany Domínguez-Pérez, Ana Matos, Guillermin Agüero-Chapin, Hugo Osório, Vitor Vasconcelos, Alexandre Campos, Agostinho Antunes

**Affiliations:** 1 CIIMAR/CIMAR—Interdisciplinary Centre of Marine and Environmental Research, University of Porto, 4450-208 Porto, Portugal; danielaalmeida23@gmail.com (D.A.); danydguezperez@gmail.com (D.D.-P.); anabastosmatos@gmail.com (A.M.); gaguero@gmail.com (G.A.-C.); vmvascon@fc.up.pt (V.V.); acampos@ciimar.up.pt (A.C.); 2 Biology Department of the Faculty of Sciences, University of Porto, 4169-007 Porto, Portugal; 3 i3S—Instituto de Investigação e Inovação em Saúde-i3S, University of Porto, 4200-135 Porto, Portugal; hosorio@i3s.up.pt; 4 Ipatimup—Institute of Molecular Pathology and Immunology of the University of Porto, University of Porto, 4200-135 Porto, Portugal; 5 Department of Pathology and Oncology of the Faculty of Medicine, University of Porto, 4200-319 Porto, Portugal

**Keywords:** cephalopods, common octopus, shotgun-proteomics, AMPs, toxins

## Abstract

Cephalopods, successful predators, can use a mixture of substances to subdue their prey, becoming interesting sources of bioactive compounds. In addition to neurotoxins and enzymes, the presence of antimicrobial compounds has been reported. Recently, the transcriptome and the whole proteome of the *Octopus vulgaris* salivary apparatus were released, but the role of some compounds—e.g., histones, antimicrobial peptides (AMPs), and toxins—remains unclear. Herein, we profiled the proteome of the posterior salivary glands (PSGs) of *O. vulgaris* using two sample preparation protocols combined with a shotgun-proteomics approach. Protein identification was performed against a composite database comprising data from the UniProtKB, all transcriptomes available from the cephalopods’ PSGs, and a comprehensive non-redundant AMPs database. Out of the 10,075 proteins clustered in 1868 protein groups, 90 clusters corresponded to venom protein toxin families. Additionally, we detected putative AMPs clustered with histones previously found as abundant proteins in the saliva of *O. vulgaris*. Some of these histones, such as H2A and H2B, are involved in systemic inflammatory responses and their antimicrobial effects have been demonstrated. These results not only confirm the production of enzymes and toxins by the *O. vulgaris* PSGs but also suggest their involvement in the first line of defense against microbes.

## 1. Introduction

Cephalopods (octopus, squid, cuttlefish, and *Nautilus*) are highly competitive and efficient predators, sharing such remarkable morphological and physiological innovations as their highly advanced visual and nervous systems, camouflage abilities, diversified body shapes, sizes, and metabolic rates [1,2]. Considering the number of species, Cephalopoda is the third richest class of the Phylum Mollusca, having around 845 extant invertebrate species [3], worldwide distributed, from shallow to deep waters [4,5,6,7,8,9]. The highest competitiveness associated to cephalopods relies mostly on their complex behavior combined with their capacity to produce venom and enzymes present in their salivary glands [10,11,12].

The absence of an external protective shell combined with the active predatory lifestyle of Coleoid cephalopods might be driving the venom evolution in those glands [13]. Indeed, the posterior salivary glands (PSGs), also known as venom glands, are actively used in feeding by cephalopods such as squids, octopuses, and cuttlefishes to subdue their preys through the inoculation of toxins [14,15,16,17]. Currently, cutting-edge approaches such as shotgun proteomics and transcriptomics have substantially increased the knowledge about those gland metabolites [18,19,20,21,22,23,24,25,26].

Fingerhut et al. (2018) took an important step in this direction combining the transcriptome of PSGs from *O. vulgaris* with the proteome of PSGs, anterior salivary glands, and saliva from adults, as well as, with the proteome of PSGs from juveniles *O. vulgaris* [22]. This study described the presence of degradative proteins and neurotoxins in the salivary apparatus of *O. vulgaris* and related species [16,20,27,28]. However, a high percentage of the detected proteins by high-throughput approaches is still annotated as “predicted” or “uncharacterized” in public databases. Therefore, their biological role in the salivary apparatus needs to be uncovered.

Cephalopod toxins repertoire comprises SE-cephalotoxin (Decapodiformes restricted), tachykinin-like peptides, “cysteine-rich secretory proteins, antigen 5, and pathogenesis-related 1 proteins superfamily members” (CAP), hyaluronidases, chitinases, phospholipase A2 (PLA_2_), carboxypeptidases, serine proteases, pacifastin peptide precursors, and DNase [13,16,17,18,19]. Besides, some organic compounds, as tetrodotoxin (TTX) and TTX-like proteins produced by endosymbiotic bacteria of the genus *Vibrio*, are used by several cephalopods to neutralize preys or as a defense against predators [13,20,29]. All venom components, including enzymes, neurotoxins and non-proteinaceous compounds seem to act in a cooperative manner to degrade membranes and tissues, facilitating the access of neurotoxins to the synaptic targets [30,31,32]. For instance, the highly toxic TTX has been found as one major components of the venom secretion in the blue-ringed octopus *Hapalochlaena* sp. [33]. It has been suggested that some venom components like enzymes (e.g., proteases and hyaluronidases) can facilitate the diffusion of the TTX into the prey tissues [19].

PSGs represent a source of underexplored bioactive compounds, including novel peptide classes like AMPs that may be involved in the first line of defense against microbes [34,35]. In fact, it is likely that octopus exploits secreted AMPs as part of an innate defense mechanism [36,37,38,39]. However, in *O. vulgaris*, those compounds remain to be fully characterized; so far only the novel peptide OctoPartenopin from the suckers has been characterized [36]. Despite the huge diversity present among AMPs, some of them, such as cationic AMPs (CAMPs), share structural features with some histones or their derivatives [40,41,42,43]. Interestingly, some classes of histones have been reported as highly expressed in the salivary secretion of *O. vulgaris* [22], but its presence in the saliva remains unclear.

**Figure 1:**
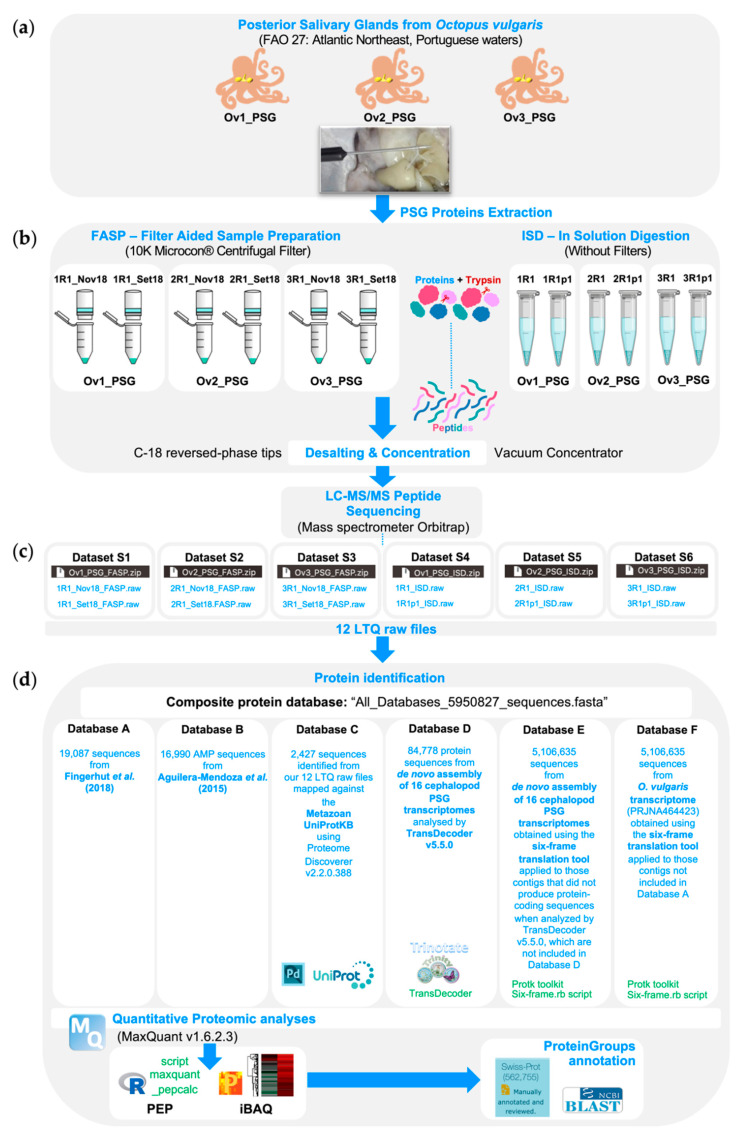
Workflow of the methodologies used to profile the whole proteome of the posterior salivary glands from the cephalopod *Octopus vulgaris*: (**a**) Sampling, dissection, and protein extraction of the posterior salivary glands (PSGs) from three adult specimens of *Octopus vulgaris* (Ov) corresponding to three biological replicates (Ov1_PSG, Ov2_PSG, Ov3_PSG); (**b**) Sample preparation protocols for LC-MS/MS analyses (FASP: Filter Aided Sample Preparation and ISD: In solution digestion), and the corresponding two technical replicates for each specimen (biological replicate); (**c**) Resulting 12 LTQ raw files from LC-MS/MS peptide sequencing using a nanoLC-MS/MS, composed by an Ultimate 3000 liquid chromatography system coupled to a Q-Exactive Hybrid Quadrupole-Orbitrap mass spectrometer (Thermo Scientific, Bremen, Germany); (**d**) Protein identification and quantitative analyses (MaxQuant freeware v1.6.2.3), downstream analyses (Perseus v1.6.2.3) and leading proteins annotation (BLASTp program against UniProtKB/Swiss-Prot and nr database).

Therefore, in this study (Figure 1), we aim to provide additional insights about the venomous repertoire of PSGs from *O. vulgaris*, as well as to unravel putative AMPs production. Thus, this study intends to be the first step for further in vitro tests to assess its potential antimicrobial activity. For this purpose, we profiled the PSGs proteome of three common *Octopus* specimens captured in the Portuguese coast; using two protein sample preparation protocols for shotgun proteomics analyses. Considering that AMPs are part of the first line of defense against microbes and virus, and that these immune components remain unexplored [34], we mapped our mass spectrometry raw data against the most comprehensive and non-redundant AMPs database published so far [44]. We dedicated special attention to histones’ classes with known antimicrobial activity [40,41,45], as well as to detect CAMPs sharing essential features with the histones [40] that were highly expressed both in the gland and saliva proteome of *O. vulgaris* [22]. Herein, we have identified most of the toxin’s families previously reported in PSGs of *O. vulgaris*. Likewise, the outcomes of this work shed new light on the PSGs functions, which might be involved in the immune responses through the production of putative histones-related AMPs.

## 2. Results and Discussion

### 2.1. LC-MS/MS Analyses and Protein Identification

The resulting 12 LTQ raw files from LC-MS/MS analyses are provided as Supplementary Material (Dataset S1–S6), deposited at the Mendeley Data repository (i.e., 2 raw files per Dataset). Overall, the strategy employed in this study (Figure 1) allowed the identification of 10,075 proteins clustered in 1868 proteinGroups (Table S1), assembled from 8704 unique peptide sequences. The term “proteinGroup(s)” is considered throughout this work to mention a cluster of proteins sharing peptide sequences identified with MaxQuant freeware.

Herein, to identify the highest possible number of peptide sequences, two sample preparation methods (FASP and ISD) were applied (Figure 1). Both methodologies yielded 8704 unique (non-redundant) peptide sequences. In total, 1133 and 8421 peptide sequences were identified with ISD and FASP protocols, respectively. Regarding the number of proteinGroups, 1824 were obtained from FASP, 447 from ISD, and 410 were identified in both protocols.

In general, FASP methodology provided better results regarding the number of proteins and peptide sequences identified. However, this approach presents some disadvantages since it is a filter-based method. In FASP protocol, samples are desalted and concentrated in the membrane of the filter by subsequent centrifugation cycles. In the present study, filters with a molecular weight cut-off of 10 kDa were applied representing a limitation to detect proteins/peptides below that molecular weight.

Although the protein digestion step can be sometimes insufficient and more prone to be affected by some contaminants present in the samples, ISD was applied as a complementary methodology to overcome FASP associated disadvantages. Despite the small number of proteins obtained with ISD, the unique and razor peptides identified with this approach increased the coverage of some proteins clustered within the above mentioned 410 proteinGroups, thus giving more accuracy to those protein reconstructions. Moreover, the ISD protocol allowed the identification of 37 exclusive clusters, including an S1 type peptidase (lcl|SRR3105321_c16_frame_6_orf_1), showed in Table S2, and presented a cluster yield of 1133 unique peptide sequences.

The original output files, obtained by MaxQuant, containing peptides’ sequences and the summary of the analyses are provided as Supplementary Material within the Dataset S7, deposited at the Mendeley Data repository. Those proteins were identified against a composite database named “All_Databases_5950827_sequences.fasta” containing 5,950,827 protein sequences composed by the data provided by the following six source databases (**Database A** to **F**). Such comprehensive database is publicly available in the corresponding *Data* article ([46]: Dataset_1): **Database A** with a total of 19,087 sequences retrieved from Fingerhut et al. (2018) [22]; **Database B**, a non-redundant AMPs database with 16,990 protein sequences from Aguilera-Mendoza et al. (2015) [44]; **Database C** made up of 2427 proteins identified with the Proteome Discoverer software v2.2.0.388 (Thermo-Fisher, Waltham, MA, USA); **Database D** with 84,778 protein sequences from de novo assembly of 16 cephalopod PSGs transcriptomes analyzed by TransDecoder v5.5.0; **Database E** with 5,106,635 protein sequences obtained with six-frame translation tool from 16 cephalopod PSG transcriptomes, which are not included in Database D; **Database F** with 720,910 protein sequences obtained with six-frame translation tool from the transcriptome of *O. vulgaris* (deposited by Fingerhut et al., 2018 [22]) but not included in Database A. Details regarding the construction of the mentioned databases, as well as the corresponding FASTA files, are provided in the associated *Data* article [46].

The number of proteins identified (10,075) were subdivided according to their source database into (Table 1): 2073 protein sequences from Database A (i.e.,: 1961 transcript sequences obtained from the *O. vulgaris* PSGs transcriptome analyzed by TransDecoder v.5.5.0; 84 transcript sequences obtained from the *O. vulgaris* PSGs transcriptome analyzed by six-frame translation tool; 28 sequences from UniProt); 44 AMPs sequences from Database B; 1845 protein sequences from Database C inferred with the six-frame translation tool from 16 cephalopods PSGs transcriptomes which were not included in Database D; 5275 transcripts of the 16 cephalopods PSGs assembled transcriptomes analyzed by TransDecoder v5.5.0 from Database D; 700 transcripts of 16 cephalopods PSGs assembled transcriptomes translated by the six-frame translation tool from Database E; 138 transcripts of *O. vulgaris* not included in Database A and analyzed by the six-frame translation tool, which integrated the Database F.

### 2.2. Comparison with a Similar Proteogenomic Study of the PSGs from O. vulgaris

Around 0.32% of the sequences, within the composite database, were retrieved from the Database A, corresponding to the third smallest contribution among the source databases (Table 1). However, the second highest percentage (20.58%) of the identified proteins (2073), by MaxQuant, was obtained from the composite database generated in this study (Table 1). From those 2073 proteins, 1759 are also leading proteins, representing 17.46% of the total of identified proteins in our analyses. Moreover, 84.85% of the proteins identified from the Database A are leading proteins, showing a qualitative measure of accuracy and significance in their identification (Table 1).

**Figure 2:**
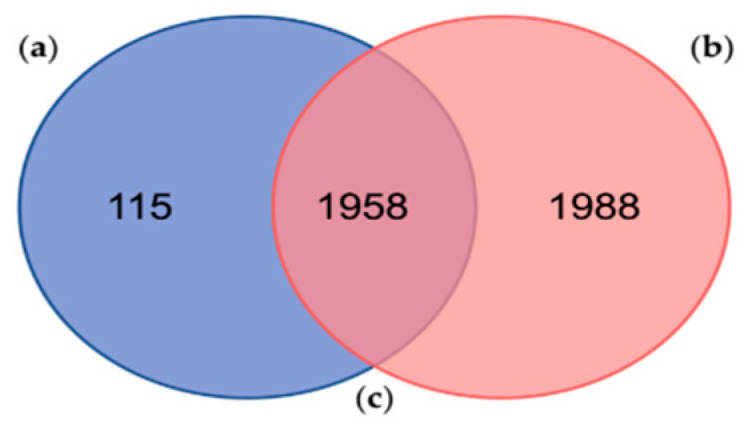
Comparison between the proteins identified in the present study and the proteins identified in a similar proteogenomic analyses of the posterior salivary glands from the cephalopod *Octopus vulgaris*: (**a**) Number of proteins identified in this study against the Database A (in blue); (**b**) Number of proteins previously identified by Fingerhut et al. (2018) against the sequences contained in the Database A, in a similar proteogenomic analyses of *Octopus vulgaris* posterior salivary glands (in red); (**c**) Number of proteins identified in both studies (shared proteins in the middle).

Fingerhut et al. (2018) found 3946 protein sequences clustered into 2810 proteinGroups using the MaxQuant freeware against its custom protein database [22], which in fact corresponds to the data contained within Database A of the present study. Out of the 3946 identified proteins by Fingerhut et al. (2018) [22], 176 protein sequences had significant hits against a custom database (UniProt_venom_and_toxin_26_06_2018) by using Protein Basic Local Alignment Search Tool (BLASTp) searches. Comparing both results, our analysis reported 115 exclusive proteins (Figure 2 and Table S3) and 1958 shared proteins with the one performed by Fingerhut et al. (2018) [22].

The number of proteins identified by Fingerhut et al. (2018) [22] was relatively higher, considering the number of proteinGroups and non-shared proteins (Figure 2). Among them, Fingerhut et al. (2018) identified a total of 116 protein sequences with homology to venom protein families, of which 95 protein sequences (Table S1) were also found in 64 of our proteinGroups (Table S4). In addition, most of these venom proteins, identified against the Database A, were leading proteins, except for the protein “lcl|TRINITY_DN26408_c0_g1::TRINITY_DN26408_c0_g1_i1::g.15697::m.15697” that belongs to the proteinGroup no. 1398 (Table S4). These results display a high reproducibility among proteogenomic studies, showing a very consistent venomous repertoire in the PSGs proteome from *O. vulgaris*. However, in general, the number of peptides identified as toxins was higher in the previous study. These results obtained by Fingerhut et al. (2018) [22] may be explained firstly by the well-executed methodology/strategy and, secondly, by the protein database used, built from the transcriptome of the same samples profiled in the proteomic analyses [22].

The strategy in applying a composite database compared to the exclusive use of the Database A (Figure 1) allowed to increase the number of the identified proteins (Table 1), to validate most of the venom protein families (Tables S2 and S4), and to improve the identification of some leading proteins (i.e., proteinGroup no. 1389, led by the protein tr|A0A0L8IA05_PD, Tables S2 and S4). Moreover, 26 additional clusters, encompassing known venom proteins and venom-like compounds, were retrieved from the other gathered databases (Table S2). Among these clusters, 13 are related to serine proteases family, 7 of which corresponded to S1 type peptidases, whereas 5 proteinsGroups belonged to serine proteases inhibitors, 4 protein clusters to metalloproteases, and other 4 clusters made up of CAP proteins (Table S2).

### 2.3. Leading Proteins Found with Homology to Venom Protein Families

**Figure 3:**
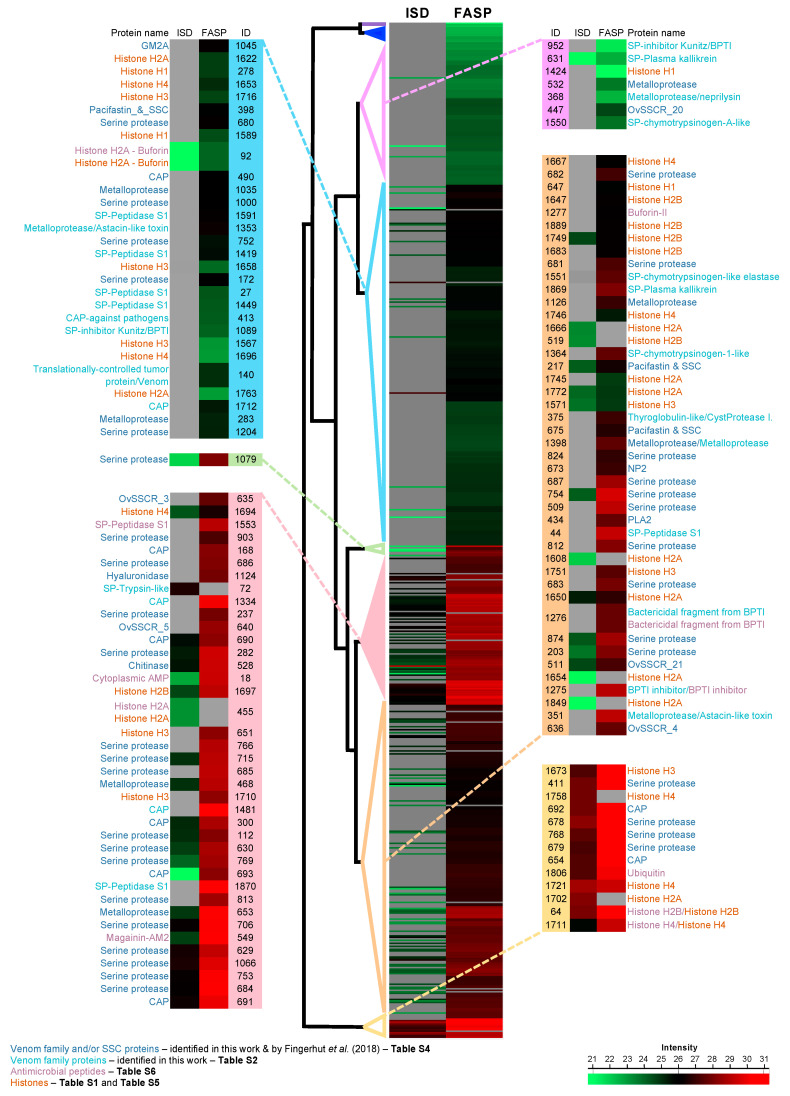
Heatmap showing proteinGroups abundance within the proteome of the posterior salivary glands from the cephalopod *Octopus vulgaris*. The composite figure of heatmaps from the *Octopus vulgaris* posterior salivary glands was built with the freeware Perseus v1.6.2.3. Cell colors show the log normalized intensity for each proteinGroup based on the absolute protein abundance using the intensity-based absolute quantification (iBAQ) score calculated by MaxQuant v1.6.2.3. The proteinGroup abundance (rows) is represented by an intensity value (see the bar scale “Intensity” on the bottom right corner) ranging from green (low protein expression) to red (more abundant proteins) for each sample preparation protocol (columns): in solution digestion (ISD) and filter aided sample preparation (FASP). Gray cells indicate that the protein was not observed in that sample. Dendrograms show hierarchical clustering of all proteinGroups as shown in the centered heatmap. The heatmaps at both sides highlight the abundance of the proteinGroups of interest (represented by ID columns), corresponding to the toxins shared with the study published by Fingerhut et al., 2018 (Venom family or SSC—Short Secreted Cysteine proteins) shown in font color blue, the venom family identified in this work (venom family proteins) in font color green water, antimicrobial peptides in font color purple and histones in font color brown. The details about the included proteins can be found in Supplementary Tables S1–S6.

Overall, we found a total of 90 proteinGroups encompassing most of the known venom protein families reported for the PSGs from O. vulgaris (Tables S2 and S4). As previously described [18,19,20,22], the venomous repertoire is composed by serine proteases, CAP, metalloproteases, serine proteases inhibitors, secreted phospholipase A (sPLA2), chitinases, hyaluronidases, pacifastin, and others SSCRs—short secreted cysteine rich proteins (Tables S2 and S4). Regarding the number of clusters, the most represented were the serine proteases family with 49 clusters accounting for 54% of the total of proteinGroups related to venom proteins, while CAP and metalloproteases accounted for 12% and 11% of the proteinGroups, respectively (Tables S2 and S4). Similarly, to previous studies [22], the most abundant venom family, considering proteinGroups intensity Based Absolute Quantification (iBAQ) scores, were serine proteases and CAP families, and to a lesser extent, metalloproteases and serine proteases inhibitors (Figure 3).

The venomous repertoire of the PSGs from *O. vulgaris* seems to be very reproducible [22] and, in general, consistent with the role proposed for this gland in cephalopods [14,16,18,19,20,22]. Serine proteases (peptidase S1 family) are widely distributed among venomous animals [47], being particularly diverse (Tables S2 and S4) and abundant (Figure 3) within the salivary gland and saliva proteome of *O. vulgaris* [22]. The presence of serine proteases in the PSGs has been associated with the external digestion of the prey after the venom inoculation [18,48], but also as an anticoagulant agent to facilitate the diffusion of other toxins into tissues [18,20,47].

We also found 12 proteinGroups whose leading protein belongs to CAP superfamily (Tables S2 and S4). This superfamily of proteins is widely present along the tree of life. Among several venomous animals, they can also be found in cephalopods, being involved in a wide range of envenomation strategies. Notwithstanding, its biological function in venoms has not been fully elucidated [47]. As a hypothesis, it has been suggested the participation of CAP protein superfamily in strategies involving the prey homeostasis disruption by the blockage of cyclic nucleotide-gated and voltage-gated ion channels and by the inhibition of smooth-muscle contraction [49].

Other abundant enzymes in the PSGs proteome were metalloproteases, represented by 11 proteinGroups (Tables S2 and S4). In snakes’ venom, metalloproteases play significant roles in bleeding, intravascular clotting, edema, inflammation, and necrosis [50], but these effects are not well studied in the PSGs from cephalopods [20,22,47]. Snakes metalloproteases act essentially by degrading the components of basement membranes underlying capillary endothelial cells, thus causing the disruption of vessel walls [51,52,53]. In *O. vulgaris*, its proteolytic effect may interfere with the hemostatic system of preys or facilitate the diffusion of other venom components promoted by its capacity of breakdown the extracellular matrix [54].

As expected, we also found chitinases, which are ubiquitous enzymes in saliva and salivary glands of cephalopods [19,22]. Chitinases are chitin degrading enzymes associated with external digestion in a wide range of organisms from fungi to humans [13,55,56], but are also associated to host infection by chitinase producing bacteria [56]. However, out of the two chitinases identified previously in the salivary proteome from *O. vulgaris*, only one seems to be present in the adults PSGs [22]. Similar to the aforementioned study, the “lcl|TRINITY_DN12896_c1_g1::TRINITY_DN12896_c1_g1_i1::g.9278::m.9278” sequence, which is the leading protein of the proteinGroup 528, was identified (Table S4), whereas the other chitinase “TRINITY_DN12584_c0_g1_i1” remains restricted to saliva and paralarvae PSGs [22]. In octopus, their activity in the venom is suggested to facilitate envenomation, leading to the damage of the prey (i.e., crustaceans) [13,19], but the functional differences between these two chitinases still need to be clarified.

Likewise, the hyaluronidases recruited into the venom act as diffusion factors across tissues, through hydrolysis of specific peptide bonds, enhancing its permeability and thus facilitating the spreading of toxins or hemostatic factors [47]. In this study, we only found one hyaluronidase cluster corresponding to the proteinGroup 1124 (Tables S1 and S4), being the same as previously reported in the PSGs from the common octopus [22]. Nonetheless, unlike *O. vulgaris*, hyaluronidases were found as particularly abundant in PSGs of southern blue-ringed octopus, *Hapalochlaena maculosa*, where they are suggested to facilitate the rapid dispersal of TTX (a potent and effective killing neurotoxin) throughout tissues [19].

Besides, in the proteinGroup 434 was identified the same and unique secreted phospholipase A_2_ (sPLA_2_), reported previously in PSGs of cephalopods [22]. Unlike serine proteases that appear highly diversified in octopods [18,19,22], sPLA_2_s seem constrained to a group of homologues sequences identified in: the octopods *O. vulgaris* [22], *Octopus kaurna*, *H. maculosa* [19]; the cuttlefishes *Sepia latimanus*, *Sepia pharaonic*; the squids *Sepioteuthis australis* and *Loliolus noctiluca* [18]. Phospholipase A_2_ (PLA_2_) enzymes hydrolyze glycerophospholipids into lysophospholipids and fatty acids producing several effects in envenomation that include antiplatelet, myotoxic, neurotoxicity, cardiotoxicity, anticoagulant, and hemolytic activities [47,57]. In octopods, sPLA_2_s have not been proved to be a particularly important component of the venom [58]; thus, their presence is still intriguing since they could be implicated in envenomation, or in the digestion of the prey, as suggested of venomous snakes [57].

Serine proteases inhibitors were also found in 5 proteinGroups (Table S2), as part of the venom components. Serine proteases are found as important venoms complement in different Phyla (e.g., cnidaria, insects, and snakes). For instance, sea anemones contain a range of Kunitz-type protease inhibitors with a dual effect, acting as a neurotoxin and as a protease inhibitor to prevent the rapid degradation of the toxins injected into prey animals or predators [59,60,61,62]. In snakes’ [63,64,65,66,67] and bees’ venoms [68], these proteins have shown antifebrin(ogen)olytic activities, acting in a cooperative manner with other venom components (e.g., serine proteases) to promote the spread of the venom [68]. In general, these mechanisms may involve serine proteases inhibitor as an anti-bleeding agent at the sting site of victims [63,64,68]. Interestingly, the leading protein of the proteinGroup 375, “lcl|TRINITY_DN12299_c0_g1::TRINITY_DN12299_c0_g1_i2::g.8022::m.8022,” previously reported in the PSGs proteome of *O. vulgaris*, had 42.5% of pairwise identity to saxiphilin-like from the snail *Pomacea canaliculate* (with NCBI accession XP_025083285.1), 30.6% to equistatin (Uniprot: P81439) from the sea anemone *Actinia equina*, and 25.4% to the toxin U24-ctenitoxin-Pn1a (Uniprot: P84032) from the spider *Phoneutria nigriventer* (Table S5). These three proteins share the thyroglobulin type I domain, proposed as inhibitors of cysteine proteases [69]. Indeed, equistatin is a secreted protein classified as a potent inhibitor of papain-like cysteine proteinases [70]. Hence, the protein found in this study could have a similar function to equistatin, as a secreted protein, since it has their corresponding signal peptide (Table S6). On the other hand, two leading proteins, tr|Q7M312_PD and sp|P00974_PD, related to serine proteases inhibitors found in this study (Table S2), were clustered with AMPs in the proteinGroups 1275 and 1276 (Table S6), respectively. Both proteins possess a Kunitz/Bovine pancreatic trypsin inhibitor domain, showing homology to aprotinin (Tables S5 and S6).

Pacifastins found in 3 protein Groups (Table S4), also meet the requirements as serine proteases inhibitor. Pacifastin is a cysteine-rich low molecular weight heterodimeric serine protease inhibitor that has an unique compact globular folding within the group of the ‘canonical’ protease inhibitors [71]. In addition to their principal function, i.e., inhibitory activity towards serine proteases, the pacifastin family presents an inhibitory action on high voltage-activated calcium channels, having been reported to have a structural similarity with the Ca^2+^ channel inhibitor ω-conotoxin GVIA [72,73]. Nonetheless, their biological functions are probably related with distinct physiological processes, namely, immune response and defense against microbial/fungal proteases [71]. Unlike the saliva fluids, pacifastin was found highly expressed in the PSGs from *O. vulgaris* [22].

Other minor components related to venom were grouped into SSCRs comprising seven proteinGroups (Table S4). Among these proteins, it was detected the protein NP2 of unknown function (proteinGroup 673, Table S4), previously identified in the PSGs from *O. vulgaris* [22] and *H. maculosa* [20]. Besides, a Ganglioside GM2 activator, previously reported in the PSGs of *O. vulgaris*, was identified in the proteinGroup 1045 (Table S4) [22]. Gangliosides are sialylated glycolipids that act as receptors for pathogenic bacterial infection on the gut epithelial cell [74]. 

In addition, the leading protein of the proteinGroup 140 (Table S2) showed homology to a translationally-controlled tumor protein, a venom protein that causes edema, obtained from the venom-gland transcriptome of the eastern coral snake *Micrurus fulvius* [75]. The translationally-controlled tumor from *M. fulvius* causes edema, enhances vascular permeability, and it is likely related to the inflammatory activity of the venom [75].

### 2.4. Histones and Antimicrobial Peptides Found in the Proteome of the Psgs from Octopus vulgaris

A total of 190 proteins clustered in 39 proteinGroups corresponded to histones (Tables S1 and S5). Previously, it was reported that histones are the most expressed proteins in *O. vulgaris* salivary apparatus [22], as expected in most tissues having a universal role in packaging DNA [76,77,78]. Histones are DNA binding proteins participating in the nucleosomes wrap inside the nucleus. However, they are present in the cytoplasm and extracellular fluids in many animal species, including fish, where they show antimicrobial activity against bacteria, viruses, parasites, and fungi [45,79,80]. They also act as neutrophil extracellular traps or mediators to bacterial killing and inflammatory responses.

Interestingly, in this work we identified 14 histones clustered in eight proteinGroups (Table S7) in the *O. vulgaris* saliva, of which three proteinGroups also comprise AMPs. Histones had already been detected as the most expressed protein in *O. vulgaris* saliva [22], despite mitosis are less significant in this fluid. It is noteworthy that some classes of histones, once secreted, may enhance an antimicrobial effect against pathogens [40], e.g., both H2A and H2B possess the capacity to neutralize endotoxins, possibly being part of the host defense barrier in the salivary glands of common octopus. This hypothesis is reinforced by the presence of AMPs in proteinGroups led by histones.

#### Are Amps and Histones Part of the Host Defense Barrier in the Salivary Apparatus of the *Octopus vulgaris*?

AMPs are widely distributed among the prokaryotes, vertebrates, invertebrates, and plants, constituting the first line of defense against microbes, and are considered part of their primary immune system [81,82]. AMPs show a great diversity, being grouped according to their structure, activity, mode of action and even according to their genetic origin [81,83]. AMP’s structural diversity can be categorized by the amino acid composition (cationic, anionic and amphipathic) and by the adopted secondary structure (α-helix, β-sheet, and extended AMPs) [83]; they commonly form amphipathic structures enabling their interaction with bacterial cell walls and their insertion into the phospholipidic membrane [84].

CAMPs and histones, mostly made up of basic and hydrophobic amino acids, are deemed to form amphiphilic three-dimensional structures able to interact with cell membranes [41]. A high number of them were detected/clustered together in the same proteinGroup by the MaxQuant (Tables S1 and S6).

Overall, the detection of 44 AMPs in 10 (Table S6) out of the 1868 proteinGroups (Table S1) shed light on the putative production by *O. vulgaris* PSGs of such compounds as part of the primary immune system. Moreover, most of the proteinGroups clustered with AMPs showed a relative high abundance (Figure 3), led by histones, serine proteases inhibitors or by known AMPs such as the Buforin-II in the proteinGroup 1277 (Table S6).

As can be observed in Table S6, the buforin II, a histone-derived AMP with bactericidal action populates the proteinGroup 1277 (Table S6), forming an exclusive Buforin cluster identified with two peptides, one of which is unique (Table S1). Buforin II (21 residues), found in the proteinGroup 92 (Table S6), is a shorter-length derivative of buforin I (39 residues). The latter was first isolated from the stomach mucosa of the Asian toad *Bufo gargarizans* [85] and later detected in the gastric fluid of pigs, cattle, and humans [43]. It shares complete amino acid identity with the N-terminal region of the histone H2A. Thus, the histone H2A is a precursor of buforin I and II and they all share the cationicity as a key feature to explain both functions, DNA packing and microbicidal activity [42]. Buforin II can penetrate into cells without damaging the cell membrane to target the DNA and RNA, resulting in a rapid bacteria death [86]. In toad gastric mucosal cells, H2A is overproduced; one fraction is targeted to the nucleus and the remaining part is secreted into the gastric lumen, where it is processed to Buforin II by pepsin C isoenzymes [87]. Something similar could occur in the salivary glands of the common octopus where the histone H2A is widely detected (Tables S1 and S5) but being less abundant than Buforin II (Figure 3).

Other AMPs detected in several proteinGroups are ubiquitin-derived peptides; in particular, we have identified similarity with the ubiquitin-like AMP (cgUbiquitin) isolated from the Pacific oyster *Crassostrea gigas* [88]. Ubiquitin is a house-keeping protein, highly conserved and present in almost all living cells [89]. Ubiquitin has been primarily known as a marker for the degradation of other proteins via proteolysis [90], and as an activator of the B-cell differentiation and of the adenylate cyclase in many tissues [89,91]. Ubiquitin is covalently attached to histone H2A in the nucleus and to several cell surface receptors in the membrane [92,93]. It also shows extracellular functions as a hormone [94] and more recently its important role on antimicrobial responses of several organisms, including marine species, has been reported [95,96,97,98]. For example, cgUbiquitin isolated from the oyster *C. gigas* is active against gram-positive and negative bacteria without causing hemolysis to human red blood cells up to 100 µg/mL. It is not membrane permeable and acts through a bacteriostatic process [88]. Ubiquitin-derived AMPs or their fragments have been already identified from the bladder of rats [99], the secretions of bovine-stimulated chromaffin cells [100], the human amniotic fluid [101] and recently from oyster gill extracts [88].

Peptides similar to the bovine pancreatic trypsin inhibitor (BPTI) were also clustered in two proteinGroups. BPTI is a protease inhibitor of 58 amino acid length sharing common features with AMPs such as (*i*) small size, (*ii*) positively charged (cationic), and (*iii*) disulphide bond stabilizer. Its antimicrobial activity was demonstrated through the fungistatic action against *Saccharomyces cerevisiae* and *Candida albicans* by inhibiting magnesium uptake into cells [102]. An ortholog of the BPTI has been detected in the skin secretions of the tomato frog (*Dyscophus guineti*) where it probably plays an alternative role as AMP, especially because magainin type AMPs were not detected in skin secretions of such species, despite their role as an important defense strategy for several species of frogs [103].

Last but not least, we detected in our samples an AMP of magainin-type as leading protein, identified from the transcriptome of the salivary glands of the common octopus. Magainins are cationic α-helical peptides, 21 to 27 residues in length, isolated from the skin of the African clawed frog *Xenopus laevis* with broad spectrum activity towards gram-positive and gram-negative bacteria, fungi, protozoa, viruses, and tumor cells [104]. Particularly, our peptide query matched with an ortholog of the original magainin, found in skin secretions of the octoploid frog *Xenopus amieti* [105].

In addition to the AMPs identified among the proteinGroups, we have also found histone fragments as leader hits in 39 proteinGroups (Tables S1 and S5) that could contribute to the defensive barrier, set by the salivary glands. As we have previously mentioned, AMPs and histones or histone fragments share relevant traits accounting for the antimicrobial activity. Most of the AMPs displayed in Table S6 are CAMPs that share structural features with histones; in fact, buforin I and II, cathepsin D, parasin I, and hipposin I are derivates from the histone H2A [40].

Furthermore, antimicrobial activity for the full-length histones in all classes (H1, H2A, H2B, H3, and H4) [40] and more recently for the histone H5 from chicken erythrocytes [41,106] has been reported. However, the fragmented histones only displayed antimicrobial action for the classes H1, H2A, and H4 [40]. In agreement with this previous knowledge that supports the antimicrobial activity of the histones, the most detected histone-like peptide within the proteinGroups belongs to the H2A class in both the salivary gland tissue and the saliva fluid, including AMPs like the buforin II within such proteinGroups (Table S7). The second most abundant, histone-like peptides of the H4 class, are present in both samples and are also grouped with other AMPs like the cgUbiquitin. Lastly, histone-like peptides of class H1 were identified in both samples as well (Table S7). All this evidence provides relevant clues about the defensive role of the antimicrobial- and histone-like peptides detected in the salivary gland of the common octopus.

## 3. Materials and Methods

### 3.1. O. vulgaris Sampling and Protein Extraction

Three fresh specimens of *O. vulgaris* caught in the eastern Atlantic (Portuguese waters) acquired in the Matosinhos market (Porto, Portugal) were transported in isothermal bags to the laboratory. Subsequently, the PSGs were dissected, and 0.5 g from each gland was introduced into lysis microtubes (Lysis Tube with impact beads, Analytik Jena AG, Jena, Germany), containing the digestion buffer. A proper volume of 1 mL of SDT buffer (2% SDS, 100 mM Tris/HCl pH 7.6, 0.1 M DTT) with Protease inhibitors (PIs, 11697498001, Roche, Mannheim, Germany) was added to each 0.5 g of sample tissue. Glands tissues were disrupted and homogenized in a cold support using the SpeedMill PLUS homogenizer (Analytik Jena AG, Jena, Germany) in continuous mode (3 min, twice) and incubated overnight at room temperature. Afterwards, samples were vortexed; heated for 3 min at 95 °C and subsequently centrifuged at 16,000× *g*, for 20 min. Finally, the supernatant was collected, and the total protein concentration was estimated according to the Bradford method [107]. Samples containing the extracted proteins were stored at −20 °C.

### 3.2. Sample Preparation for LC-MS/MS Analyses

Protein samples from PSGs comprising three biological replicates were processed in duplicates following two distinct protocols (i.e., a total of six protein samples for each protocol): FASP—filter aided sample preparation [108] and ISD—in solution digestion using RapiGest SF Surfactant according to fabricant specifications from (Waters Corporation, Milford, MA, USA). More details can be found below.

#### 3.2.1. Filter Aided Sample Preparation

The extracted proteins from PSGs (30 μg) were alkylated and digested with trypsin (recombinant, proteomics grade, Roche, Basel, Switzerland), at enzyme to protein ratio of 1:100 (*w*/*w*), for 16 h at 37 °C, in centrifugal filter units with nominal molecular weight limit (NMWL) of 10 kDa (MRCPRT010, Millipore, Billerica, MA, USA). Peptides were subsequently recovered by centrifugal filtration, acidified with formic acid (FA 10%, *v*/*v*), desalted and concentrated by reversed-phase extraction (C18 Tips, 100 µL, Thermo Scientific, 87784) using acetonitrile (ACN 70%, *v*/*v*) and trifluoroacetic acid (TFA 0.1%, *v*/*v*) for peptide elution. Finally, the peptides were redissolved in 0.1% FA (*v*/*v*) in water to the concentration of 0.04–0.06 μg/μL and conducted to LC–MS/MS.

#### 3.2.2. In Solution Digestion

The extracted proteins from each replicate (200 μg) were solubilized in 50 μL of 0.1% *Rapi*Gest SF surfactant (RapiGest™SF, Waters, Milford, MA, USA) in ammonium bicarbonate 50 mM. The disulfide bonds were methylated by adding 5 μL of Dithiothreitol (DTT) solution in ammonium bicarbonate 50 mM to a final concentration of 5 mM and incubated at 60 °C for 30 min. Protein samples were kept at room temperature for 10 min and posteriorly methylated by adding 5 μL of iodoacetamide (IAA) to a final concentration of 15 mM and incubated in the dark for 30 min. Protein samples (100 μg) were alkylated and digested with trypsin (recombinant, proteomics grade, Roche, Basel, Switzerland), at enzyme to protein ratio of 1:100 (*w*/*w*), in a wet chamber for 3 h at 37 °C. Trypsin activity was inhibited by acidification with 2.5 μL of 13% TFA to reach a final concentration of 0.5% TFA (approximately pH = 2) and incubated at 37 °C for 30 min. RapiGest was removed by centrifugation at 16,000× *g*, and the supernatant containing the trypsin digestion products was centrifuged and transferred to a new microtube. The digested peptides were concentrated until removing all the solvent using a SpeedVac System composed by one CentriVap concentrator, a CentriVap −50 °C cold trap (from LABCONCO, Kansas City, MO, USA) and a vacuum pump (Welch-Ilmvac, Niles, IL USA). Finally, the peptides were redissolved in 0.1% FA (*v*/*v*) in water to the concentration of 0.04–0.06 μg/μL and conducted to LC–MS/MS.

### 3.3. LC-MS/MS Analyses

Protein samples prepared according to the above described FASP and ISD protocols were processed using a nanoLC-MS/MS, composed by an Ultimate 3000 liquid chromatography system coupled to a Q-Exactive Hybrid Quadrupole-Orbitrap mass spectrometer (Thermo Scientific, Bremen, Germany). Samples were loaded onto a trapping cartridge (Acclaim PepMap C18 100 Å, 5 mm × 300 µm i.d., 160454, Thermo Scientific, Bremen, Germany) in a mobile phase of 2% ACN, 0.1% FA at 10 µL/min. After 3 min loading, the trap column was switched in-line to a 15 cm by 75μm inner diameter EASY-Spray column (ES800, PepMap RSLC, C18, 3 μm, Thermo Scientific, Bremen, Germany) at 300 nL/min. Separation was generated by mixing A: 0.1% FA, and B: 80% ACN, with the following gradient: 5 min (2.5% B to 10% B), 60 min (10% B to 35% B), 5 min (35% B to 99% B), and 5 min (hold 99% B). Subsequently, the column was equilibrated with 2.5 % B for 12 min. Data acquisition was controlled by Xcalibur 4.0 and Tune 2.8 software (Thermo Scientific, Bremen, Germany).

The mass spectrometer was operated in data-dependent positive acquisition mode alternating between a full scan (*m*/*z* 380–1580) and subsequent HCD MS/MS of the 10 most intense peaks from full scan (normalized collision energy of 27%). ESI spray voltage was 1.9 kV and capillary temperature was 275 °C. Global settings: use lock masses best (*m*/*z* 445.12003), lock mass injection Full MS, chrom. peak width (FWHM) 15 s. Full scan settings: 70 k resolution (*m*/*z* 200), AGC target 3e6, maximum injection time 50 ms. Data-dependent settings: minimum AGC target 8e3, intensity threshold 7.3e4. Charge exclusion settings: unassigned, 1, 8, >8, peptide match preferred, exclude isotopes on, dynamic exclusion 20 s. MS2 settings: microscans 1, resolution 35 k (*m*/*z* 200), AGC target 2e5, maximum injection time 110 ms, isolation window 2.0 *m*/*z*, isolation offset 0.0 *m*/*z*, spectrum data type profile.

### 3.4. Protein Identification

#### 3.4.1. Quantitative Proteomic Analyses

In total, 12 raw files comprising three biological replicates in duplicates from the two sample preparation methods employed (deposited to the Mendeley Data repository: 6 from FASP—Dataset S1–S3 and 6 from ISD—Dataset S4–S6) were searched against a custom protein database (for more details about this database see the corresponding *Data* article [46]) using MaxQuant v1.6.2.3 freeware software [109]. MaxQuant parameters for protein identification were: MS and MS/MS tolerances of 20 ppm and 0.5 Da, respectively; two missed tryptic cleavages were allowed; PSMs were accepted at a 1% false discovery rate (FDR) and trypsin was selected for protein cleavage. Carbamidomethylation were selected as static modifications, while Oxidation of Methionine and Acetylation of protein N-terminus were chosen as variable modifications. Protein quantification was based on approximate absolute protein abundance, an iBAQ score calculated by MaxQuant. The Posterior Error Probability (PEP) of proteinGroups was calculated using the script maxquant_pepcalc, available at https://github.com/pstew/maxquant_pepcalc. Venn diagrams were used to identify the shared proteins using an online free tool, available at the webserver of the Bioinformatics and Evolutionary Genomics Center (BEG/Van de Peer Lab site, Ghent University, Belgium, http://bioinformatics.psb.ugent.be/webtools/Venn/).

#### 3.4.2. Maxquant Proteingroups Annotation

All proteinGroups obtained with MaxQuant software were annotated through the leading protein as the best hit of each cluster. The strategy employed was based on homology search using a local BLAST with BLASTp program against the “UniProtKB/Swiss-Prot, the Manually Annotated Section of the UniProt KnowledgeBase” (accessed 13 May 2019) [110], “UniProtKB/Swiss-Prot Tox-Prot” (http://www.uniprot.org/program/Toxins; accessed 13 May 2019) [111], and against the National Center for Biotechnology Information (NCBI) non-redundant protein database (nr database: ftp://ftp.ncbi.nlm.nih.gov/blast/db; accessed 25 June 2018), setting a cut-off e-value of 1e−3. When the leading protein of the proteinGroup returned no hit and/or the annotation was “uncharacterized protein”, an additional search with the BLASTp program was performed online using automatic adjustment parameters. Considering that toxins and AMPs are secreted proteins and peptides, thus signal peptides were predicted using the SignalP v5.0 server (https://services.healthtech.dtu.dk/service.php?SignalP-5.0) [112], as well as Secretory/Non-secretory pathway inference using the DeepLoc-1.0 server (http://www.cbs.dtu.dk/services/DeepLoc) [113].

## 4. Conclusions

The proteomic profile of the PSGs from *O. vulgaris* was demonstrated to be very reproducible and consistent with the previous studies [14,16,18,19,20,22]. The proteome was dominated by venom protein families, histones, and other ubiquitous cellular proteins. The venomous repertoire encompasses serine proteases, CAP, metalloproteases, serine proteases inhibitors, sPLA_2_, chitinases, hyaluronidases, pacifastin, and others SSCRs. As previously described [22], serine proteases, CAP and metalloproteases are more abundant within the venomous proteome, whereas serine protease inhibitors, sPLA_2_s, pacifastins, and other SSCRs can be either considered as complementary components for the venom function or their role should be further clarified.

The methodology employed in this study using a large protein database built from cephalopods PSGs resulted suitable for our aims and support our findings. The search engine “Andromeda” (embedded into MaxQuant freeware) translates our experimental data, from spectra to peptide sequences considering the information contained in the composite protein database provided (All_Databases_5950827_sequences.fasta file). Therefore, we increased the probabilities to detect/identify our raw data against the protein database by considering the most comprehensive proteomic database about cephalopod PSGs built so far, and by adding a non-redundant AMPs database, as an interesting approach to recover some information that usually have been discarded. Thus, this approach is suitable for a large-scale characterization of proteomes, being more useful to perform exploratory analyses of complex biological samples (presence/absence, relative abundance), than extensive qualitative characterizations such as the determination of full-length protein sequences and structures.

Although it is recommended to use a custom protein database derived from the transcriptome of the same samples, our strategy allowed us to increase the number of identified proteins by using a composite protein database. The use of a composite protein database including translated PSGs transcriptomes from cephalopods provided additional insights about toxins protein families and bioactive compounds such as putative AMPs.

Most of the proteinGroups containing AMPs showed a relative high abundance, and some of them were clustered in AMPs-exclusive proteinGroups. These AMPs were mainly related to the antimicrobial compounds such as to the ubiquitin fragment and carboxyl ribosomal protein S27 extension, histones H2B and H4, antibacterial and bactericidal fragment of BPTI, and the Buforin-II. This study sheds new light about a putative role of the cephalopods PSGs in the first line of defense against pathogens. However, both the presence of AMPs, and the high expression of histones in salivary secretion still need to be unequivocally confirmed.

## Figures and Tables

**Table 1:** Summary of the number of protein sequences gathered in the composite database according to their source database and identified by MaxQuant analyses.

Custom Databases	# Protein Sequences ^7^	% Protein Sequences Relatively To The Composite Database ^9^	# Proteins Identified ^10^	% Proteins Identified Relatively To The Total Of Proteins Identified ^12^	# Leading Proteins ^13^	% Leading Proteins Relatively To The Total Of Proteins Identified ^14^	% (# Leading Proteins Relatively To The # Proteins Identified Per Db) ^15^
**A** ^1^	19,087	0.32	2073	20.58	1759	17.46	84.85
**B** ^2^	16,990	0.03	44	0.44	12	0.12	27.27
**C** ^3^	2427	0.04	1845	18.31	1180	11.71	63.96
**D** ^4^	84,778	1.42	5275	52.36	3075	30.52	58.29
**E** ^5^	5,106,635	85.81	700	6.95	249	2.47	35.57
**F** ^6^	720,910	1.21	138	1.37	70	0.69	50.72
Total	**5,950,827** ^8^	100	**10,075** ^11^	100	6345	62.98	-

^1^ protein sequences from a database made available by Fingerhut et al. (2018) [22]. ^2^ non-redundant antimicrobial peptides from a database made available by Aguilera-Mendoza et al. (2015) [44]. ^3^ proteins identified by Proteome Discoverer v2.2.0.388. ^4^ protein sequences from *de novo* assembly of 16 transcriptomes of cephalopod PSG analyzed by TransDecoder v5.5.0. ^5^ protein sequences obtained with six-frame translation tool from 16 PSG transcriptomes, which are not included in Database D ^4^. ^6^ protein sequences obtained with six-frame translation tool from the transcriptome of *O. vulgaris* (deposited by Fingerhut et al. (2018) [22]) but not included in Database A ^1^. ^7^ This is the number (#) of protein sequences in each database. ^8^ This is the total of protein sequences present in the composite database (All_Databases_5950827_sequences.fasta), used for MaxQuant analyses, that came from each one of the six smaller source databases (A to F). ^9^ This is the percentage (%) of protein sequences from each database that is present in the composite database, i.e.,: *% =* (# **protein sequences** ^7^/**5,950,827** ^8^) × 100. ^10^ This is the number (#) of protein sequences identified by MaxQuant v1.6.2.3 in the composite database that came from each small database (A to F). ^11^ This is the total number of protein sequences identified by MaxQuant v1.6.2.3 in the composite database. ^12^ This is the percentage (%) of protein sequences identified by MaxQuant software v1.6.2.3 in the composite database that came from each smaller database (A to F), i.e.,: *% =* (**# protein identified** ^10^/**10,075** ^11^) × 100. ^13^ This is the number (#) of leading proteins identified by MaxQuant v1.6.2.3 in the composite database that came from each smaller database (A to F). ^14^ This is the percentage (%) of leading proteins identified by MaxQuant v1.6.2.3 in the composite database, that came from each smaller database (A to F), and that is present in the total of proteins identified, i.e.,: *% =* (# **leading proteins** ^13^/**10,075** ^11^) × 100. ^15^ This is the percentage (%) of leading proteins identified by MaxQuant v1.6.2.3 in the composite database, that came from each smaller database (A to F) relatively to the number of proteins identified per database (DB), i.e.,: *% =* **(# leading proteins** ^13^/**# protein identified** ^10^) × 100.

## Supplementary Materials

The following are available online at https://www.mdpi.com/2079-6382/9/11/757/s1 Table S1: MaxQuant output file of the posterior salivary gland proteome from the cephalopod *Octopus vulgaris* after filtering. Table S2: Leading proteins with homology to known venom protein families identified in this work. Table S3: Proteins identified in this study from Database A that were not previously reported at the proteomic level by Fingerhut et al. 2018. Table S4: Leading proteins ^1^ shared with those toxins-like and short secreted cysteine rich proteins previously reported by Fingerhut et al. (2018). Table S5: Leading protein annotation of the posterior salivary glands’ proteome from the cephalopod *Octopus vulgaris*. Table S6: Antimicrobial peptides found in the proteome of posterior salivary glands from the cephalopod *Octopus vulgaris*. Table S7: Histones found in the proteome of the posterior salivary glands and saliva from the cephalopod *Octopus vulgaris*. The following are available online at the Mendeley Data repository: Dataset S1: http://dx.doi.org/10.17632/csc8shzkwc.1. Dataset S2: http://dx.doi.org/10.17632/787g95ppwv.1. Dataset S3: http://dx.doi.org/10.17632/8fhx775zdf.1. Dataset S4: http://dx.doi.org/10.17632/d6wxyt22kx.1. Dataset S5: http://dx.doi.org/10.17632/zbtkf2nsvh.1. Dataset S6: http://dx.doi.org/10.17632/df4cbg73tx.1. Dataset S7: http://dx.doi.org/10.17632/j4khf8ngkb.1.
